# Behavioural Lateralization in Budgerigars Varies with the Task and the Individual

**DOI:** 10.1371/journal.pone.0082670

**Published:** 2013-12-06

**Authors:** Ingo Schiffner, Mandyam V. Srinivasan

**Affiliations:** 1 Queensland Brain Institute, University of Queensland, St Lucia, Queensland, Australia; 2 School of Information Technology and Electrical Engineering, University of Queensland, St Lucia, Queensland, Australia; 3 ARC Centre of Excellence in Vision Science, University of Queensland, St Lucia, Queensland, Australia; Utrecht University, Netherlands

## Abstract

Handedness/footedness and side biases are a well-known phenomenon in many animals, including humans. However, these so-called biases have mostly been studied at the population level - individual biases have received less attention, especially with regard to consistency over different tasks. Here we investigate behavioral lateralization in 12 male Budgerigars, *Melopsittacus undulatus*, a social parrot inhabiting the Australian bushlands. We performed 5 types of experiments to investigate lateralization, in tasks that involved climbing onto a perch, or landing on perches arranged in various configurations. The birds displayed highly significant, individually varying biases. The bias displayed by any particular individual varied with the task, in strength as well as polarity. Analysis of the data revealed that the preferred foot used for climbing did not coincide with the foot that was used while landing. Thus, landing choices are probably not determined by foot bias. Furthermore, these individual preferences were overridden completely when a bird had to perform a task simultaneously with another bird.

## Introduction

Most work to date on “handedness” and behavioural and brain lateralization has shown that animals display consistent side biases. For example, chicks are known to use the right eye preferentially for detecting and inspecting food, and the left eye for detecting predators [1]. Other studies that have found behavioural lateralization at the population level involve detection of either food or prey (cane toads: [2], domestic chick: [3]), or predators (domestic chicks: [1], fish: [4]) or social interactions with conspecifics (domestic chicks: [5], quails: [6]). 

One of the patterns emerging from these studies is that population biases seem to be present primarily in social animals. Examples include several species of Australian parrot [7-9], three species of toad [10] and several species of fish [4] and honeybees [11,12]. The underlying rationale is that unilateral handedness or footedness would direct a group of individuals of the same population to move or respond in the same direction, ensuring that all individuals stay with the group and do not become isolated and vulnerable, for example when being chased by a predator [13]. To test this notion, it is important to examine whether the biases that are displayed by individuals when they are tested singly, continue to persist when they are tested in the company of other individuals. This is one of the aims of our study.

While most studies of behavioural lateralization have reported population biases (as described above), relatively little effort has been devoted to investigating the variation of lateralization from one task to another, or to the variation of lateralization from one individual to another even with respect to a single, specific task - although these questions have persisted for more than 30 years (See open peer comments in (See open peer comments in [14 ,15,16]). Recent studies, at least in fish [17,18], aim to shed some light on this problem, but relatively little is known about individuality in birds. Thus, another aim of our study is to examine whether lateralization varies from individual to individual, and whether a given individual displays consistent lateralization across tasks that are closely related.

Here we find that, even within a single species (the Budgerigar, *Melopsittacus undulatus*), different individuals can display different biases with respect to a given task. Furthermore, the bias that is displayed by a given individual can vary dramatically from one task to another, and it can also depend upon whether the bird is tested alone or in the company of another bird that is performing the same task concurrently. This is true even when the tasks are closely related, suggesting that behavioural lateralization is more complex, intricate and variable than previously imagined.

## Methods

### Ethics Statement

All experiments were carried out in accordance with the Australian Law on the protection and welfare of laboratory animals and with the approval of the Animal Experimentation Ethics Committees of the University of Queensland, Brisbane, Australia (Permit QBI/646/07/ARC).

### Subjects

The subjects were 12 adult male Budgerigars (*Melopsittacus undulatus*) between one and five years old. They were acquired in their first year of life and were housed in an outdoor aviary. The experiments were conducted at the University of Queensland’s Pinjarra Hills Field Station. 

### Apparatus and Implementation

The experiments took place indoors in a tunnel that was 7.28 m long, 1.36 m wide and 2.44 m high. The walls and the ceiling of the tunnel had been painted white and the floor was made of grey concrete. The end walls were covered with black cloth, to standardise visual cues. Five different types of experiments were conducted to investigate whether the birds displayed side biases and footedness whilst landing on or climbing onto perches that were presented in various configurations. 

### Training

**Figure 1:**
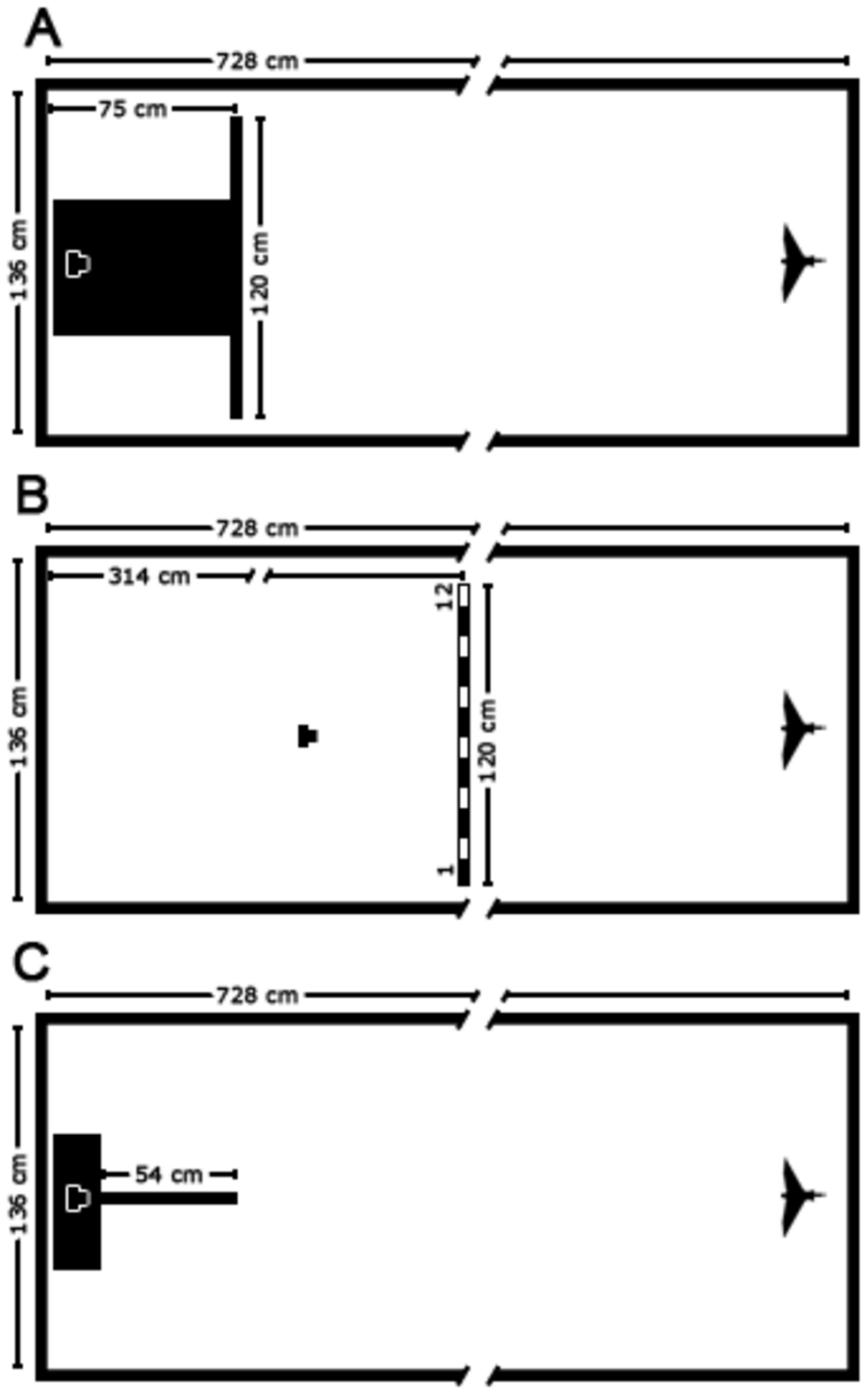
Setup of the experimental arena. Shown are the positions of the perches in relation to the scale of the tunnel (not shown in full length) for Experiment 1 (A), Experiments 2, 3 and 5 (B), and Experiment 4 (C). The starting positions of the bird, (or birds), and the position of the camera are indicated by the respective symbols. In (B) the 12 subdivisions of the long perch are shown as alternately black and white only for a clearer visualization of their geometry.

The Budgerigars were trained to take off from a hand-held wooden perch (60 cm long) and to land on one or two perches placed at various positions in the tunnel, depending upon the particular experiment (see Figure 1). The experimenter initiated the take off by rotating the perch gently about its longitudinal axis, or lowering it abruptly by a few centimetres. In general, the birds required little or no training. When necessary, training was commenced with the take-off occurring close to the test perches (about 20 cm away). This distance was then increased gradually until the birds eventually flew toward and landed on the test perch. Neither during training, nor during the experiments were the birds rewarded in any way. 

### Experiment 1 (choice between two perches)

In the first experiment the birds were offered two perches on which they could land, one to the left and the other to the right of their flight path, and their landing choices were recorded (see Figure 1 A). The twin perches were created by positioning a 120 cm long wooden rod in the middle of the tunnel, oriented perpendicularly to the side walls, at a distance of 75 cm from the back wall. The rod was supported by a box (54 cm wide and 100 cm high). A black cloth was used to cover the centre of the perch, creating the appearance of two transversely oriented, oppositely directed perches, each 33 cm long.

### Experiment 2 (free landing on a single, long perch)

In the second experiment the birds were presented with a single, transversely oriented perch 120 cm long (see Figure 1 B). The perch was suspended from the ceiling by ropes at a height of 120 cm, and the birds were free to land anywhere along its length.

### Experiment 3 (free landing on a single, long perch, two birds)

The third experimental configuration was identical to that in Experiment 2, except that two birds were released simultaneously (see Figure 1 B). The landing positions of both birds were recorded. We tested two different configurations, in a symmetrical design. In the first configuration, termed the “Ipsilateral" configuration, birds were released from the perch such that their relative positions at take off corresponded to their relative positions at landing, as derived from each bird’s mean landing position in Experiment 2, where the birds were tested individually. In the second configuration, termed the “Contralateral" configuration, the birds were released in a configuration that was the opposite of their relative positions at landing as recorded in Experiment 2. For instance, if bird A had landed at a position that was to the left of bird B, as determined by the mean landing positions of the two birds in Experiment 2, then bird A was positioned to the left of bird B in the “Ipsilateral configuration”, and to the right of bird B in the “Contralateral configuration”. For each pair of birds, we conducted 5 trials in each configuration.

### Experiment 4 (landing on a single, axially oriented perch)

In this setup the birds were presented with a single perch placed at the far end of the tunnel, oriented perpendicular to the back wall. (see Figure 1 C). The perch was supported at a height of 105 cm by a box, which was covered with a black cloth. 

### Experiment 5 (foot choice when climbing onto a perch)

In this experiment the bird sat on the suspended perch - the same perch as in Experiments 2 and 3 (see Figure 1 B). The experimenter, standing directly in front of the bird, induced the bird to climb on to a smaller perch, 60 cm long, by pressing it gently against the bird’s chest. This is a naturally elicited response, which requires no previous training. 

### Experimental Procedure

In order to prevent external factors from influencing the birds in their choices, we took care to ensure that all of the experimental configurations were perfectly symmetrical. The birds were always released at the far end of the tunnel, close to the midline. The experimenter stood behind the take-off perch (whenever possible, behind a black cloth) and held the perch horizontally with both hands at approximately the same height as the landing perch. In those cases where the perch had to be held with one hand, the right or the left hand was used alternately. In addition to these precautions, Experiments 1 and 4 were repeated with the entire setup rotated by 180 degrees (i.e. with the birds flying in the opposite direction in the tunnel), to exclude potential influences from extraneous cues, such as non-uniform illumination, acoustic noise or the geomagnetic field.

A total of 12 male Budgerigars were used in the study. We conducted twenty trials for each bird in Experiments 1, 2, 4 and 5. In Experiment 3 we released two birds simultaneously, by combining each bird with one of two or three other birds, in a total of 13 different combinations. The birds were released close to each other, with a separation not exceeding 20 cm, and with one bird positioned on the left side of the perch and the other on the right side. Each bird took off five times from the left side and five times from the right side. We only used data from flights in which the birds took off within 1 second of each other; on average, the birds took off within 15 ms and landed within 30 ms of each other.

### Analysis of Video Data

The flights of the birds were recorded at 100 frames per second using a Lightning RDT high-speed digital camera (DRS data & imaging systems, Inc., Oakland, NJ). Camera operation and video acquisition were controlled by special-purpose software (MiDAS 2.0 (Xcitex, Inc., Cambridge, MA)).

The camera was positioned at one end of the tunnel, behind the landing perch (or perches) and roughly at the same height. The camera was carried by a tripod with a rotatable 3-axis head (Manfrotto), equipped with a spirit level to aid levelling.

For each experiment, the data was recorded in each trial as follows:

In Experiment 1 we recorded whether the bird landed on the left-hand perch or the right-hand perch, and which foot was used to make first contact. In Experiments 2 and 3 the landing perch -120 cm long - was subdivided into 12 equal segments, with Segment 1 being the leftmost segment, and Segment 12 the segment furthest to the right (see also Figure 1 B). In Experiment 2 we recorded the segment at which the bird landed. In Experiment 3, which involved releasing two birds simultaneously, we also recorded the starting position of each bird (left or right). If the bird started or landed on the boundary between two segments, the position was reckoned to be the mean of the values for the two segments. In Experiment 4 we recorded whether the bird approached the axially-oriented perch from the left or from the right to land on it, and which foot was used to make first contact. And in Experiment 5 we recorded which foot the bird used first to step on to the perch that was held against its chest. 

### Statistics

We used the Sign test [19] In order to check for significant preferences on the individual level (N=20 trials per bird) and for biases at the level of the entire population (n=12 birds). Second order correlations between the individual measures of preference were performed using the Spearman rank correlation [20]. To test for the existence of correlations between multiple measurements of preference we used canonical correlation analysis [21]

## Results

### Experiment 1 (choice between two perches)

**Figure 2:**
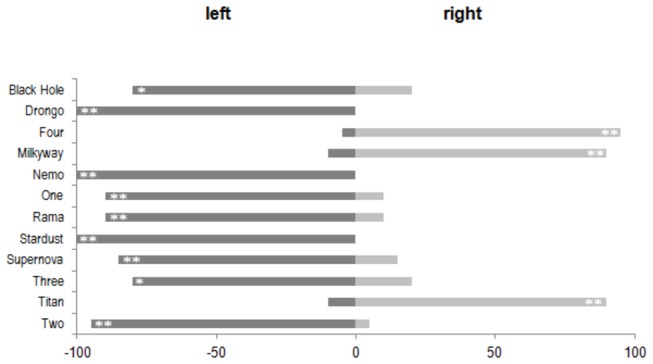
Choice between two perches for landing (Experiment 1). Frequencies of landings on the left-hand or the right-hand perch. Significance levels for the individual preferences are indicated by asterisks (**: p < 0.01; *: p < 0.05; +: p < 0.10).

The birds showed a strong, individual-dependent preference for landing on one particular perch. As shown in Figure 2, each bird chose the perch on its “preferred” side with a frequency between 80 % and 100 %. This preference was statistically significant for each bird (Sign test p < 0.05). Nine birds preferred the left-hand perch, and three birds the perch on the right, indicating a tendency for an overall population bias towards the left (Sign test p < 0.10). 

### Experiment 2 (free landing on a single, long perch)

**Figure 3:**
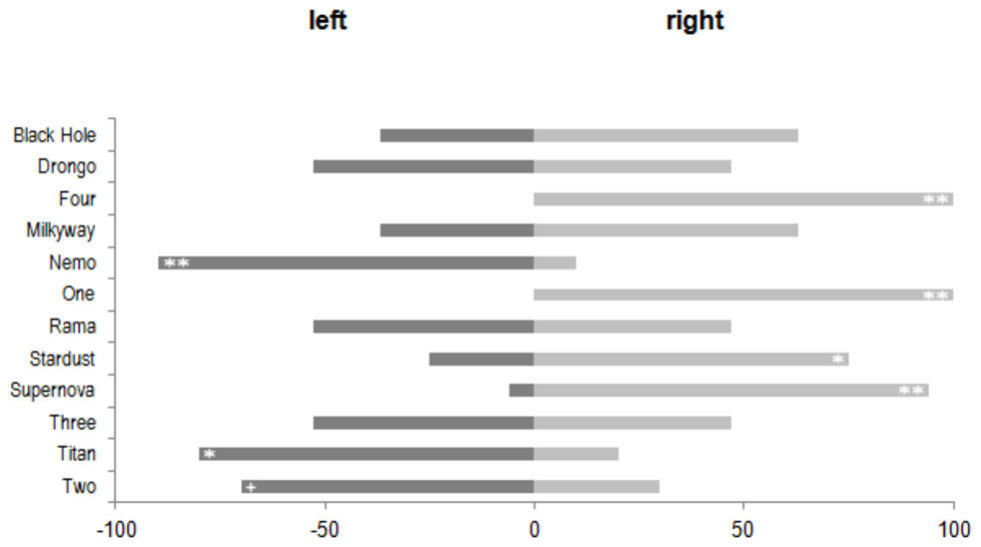
Free landing on a single long perch (Experiment 2). Frequencies of landings on the left or right side of the perch. Significance levels for the individual preferences are indicated by the following symbols (**: p < 0.01; *: p < 0.05; +: p < 0.10).

Experiment 2 revealed that the distribution of the landing positions differed substantially from bird to bird. (Histograms of the landing positions for each bird are shown in Figure S1 and the mean landing positions and the standard deviations are given in Table S1). Only two birds showed a preference for one specific location on the perch. The others showed an almost uniform distribution of landing positions, spread over a much larger portion of the perch. When considering only whether the birds landed on the right or left side of the perch, and excluding landings in the central segment, we found that only half of the birds exhibited a significant preference for one side (Sign test p < 0.05; See Figure 3). Even more intriguingly, four birds displayed a side bias that was exactly the opposite of what they displayed in Experiment 1. Overall the number of left and right biased birds was equal, thus no population bias was observed in this task (Sign test p > 0.10). 

 As an aside, it is worth noting that if we divide the landing perch into a broad central region comprising segments 5-8, and two outer regions comprising segments 1-4 and 9-12, there was an overall significant preference for landing in the central region (Sign test p < 0.05).

### Experiment 3 (free landing on a single, long perch, two birds)

In this experiment two birds were released simultaneously, and their landing positions were recorded in the same way as in Experiment 2. From this data we determined whether the landing birds had retained their take-off configuration, or had swapped positions (i.e. crossed over). Table 1 shows the number of times that crossovers occurred between take-off and landing, for the Ipsilateral configuration and the Contraleral configuration. Note that, for each pair of birds, the number of crossovers can range between a maximum of five and a minimum of zero. If crossovers occurred randomly, we would expect the mean crossover frequency to be 50%. We observe from Table 1, however, that the mean crossover frequency was significantly lower than this value, being 15% for the Ipsilateral configuration (Sign test p < 0.05) and 11% for the Contralateral configuration (Sign test p < 0.01). This means that, in each condition, the birds were more likely to retain their initial configuration during the entire flight, and less likely to cross over. There were no indications that any bird, when released on its preferred side (Ipsilateral configuration) showed an increased tendency to land closer to its preferred landing location (Wilcoxon Signed Rank Test; T=22; p > 0.10; see also Table S2 Supplemental Material). This is true even for the bird that landed first on the perch (Wilcoxon Signed Rank Test; T=31; p > 0.10). Similarly, the temporal sequence in which the birds took off was maintained during landing. In other words, in most of the trials, the bird that took off first was also the first bird to land. The frequency of overtaking manoeuvres was 30% or lower for all thirteen pairs of birds that were tested, and this frequency was significantly lower than that expected by chance (50%; Sign test p < 0.01; see Table 1).

**Table 1:** Frequencies of crossovers/overtaking manoeuvres.

	**Frequency of crossovers/overtaking manoeuvres for all pairs of birds**						
	**Blackhole**	**Blackhole**	**Drongo**	**Drongo**	**Nemo**	**Nemo**	**One**
**Configuration**	**Milkyway**	**Supernova**	**Four**	**Three**	**Four**	**Two**	**Rama**
**Ipsilateral**	0%	0%	0%	0%	0%	0%	20%
**Contralateral**	80%	0%	40%	0%	20%	0%	0%
**Overtaking**	0%	10%	0%	20%	20%	10%	0%
	**One**	**One**	**Three**	**Three**	**Stardust**	**Stardust**	
**Configuration**	**Four**	**Two**	**Rama**	**Two**	**Supernova**	**Titan**	**Mean**
**Ipsilateral**	0%	60%	0%	60%	0%	60%	15%
**Contralateral**	0%	0%	0%	0%	0%	0%	11%
**Overtaking**	10%	0%	0%	0%	0%	30%	8%

### Experiment 4 (landing on a single, axially oriented single perch)

**Figure 4:**
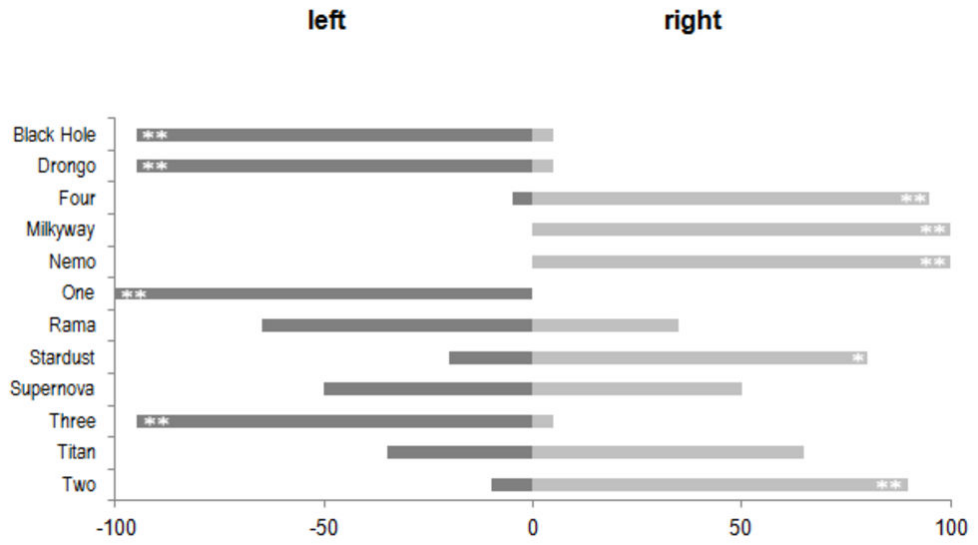
Landing on a single, axially oriented perch (Experiment 4). Frequencies of landings from the left or the right side. Significance levels for the individual preferences are indicated by the following symbols (**: p < 0.01; *: p < 0.05; +: p < 0.10).

In this experiment we investigated the behaviour of birds when they approached a perch that was oriented parallel to their flight path, requiring them to land on it by either approaching it from the left and turning right, or vice versa. The results of this experiment again differ from those of Experiment 1 (see Figure 4). All except three birds showed strong, individual biases, with frequencies of approach from a particular side ranging between 80% and 100 % (Sign test, p < 0.05). Here too the number of right and left biased birds is almost the same (left: 5; right: 6; undetermined: 1), thus not indicating the presence of an overall population bias (Sign test p > 0.10).

### Experiment 5 (foot choice when climbing onto a perch)

**Figure 5:**
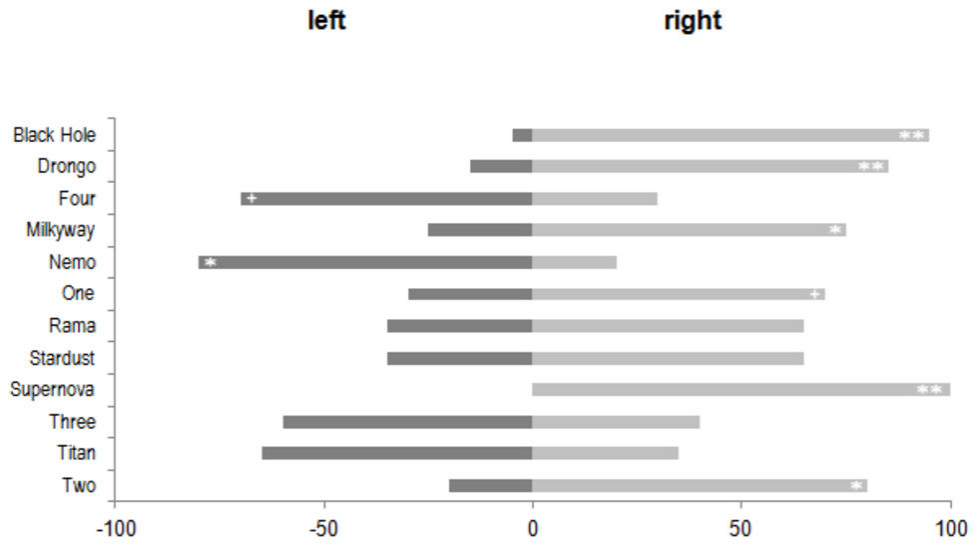
Foot choice when climbing onto a perch (Experiment 5). Frequencies of use of left or right foot. Significance levels for the individual preferences are indicated by the following symbols (**: p < 0.01; *: p < 0.05; +: p < 0.10).

Six of the birds showed a significant preference for initiating the climb with a specific foot (Figure 5; Sign test p < 0.05). Five of them preferred the right foot and only one preferred the left foot. Two additional birds showed a non-significant tendency to prefer a specific foot (Sign test p < 0.10). Eight birds preferred to use the right foot, and four birds preferred to use the left foot. No overall population bias was observed in this task, either (Sign test p < 0.10). 

### Foot Preference during Landing

In Experiments 1 and 4 we also looked at which foot the birds used to make first contact with the perch while landing. In Experiment 1 the birds showed a clear preference to touch down with the foot opposite to the direction of approach (i.e. foot facing away from the wall), namely, the left foot when landing on the right-hand perch and the right foot when landing on the left-hand perch (all birds 84-100%; all Sign test p < 0.01, except for one bird with too few observations; see Table S3 in the Material for details). In Experiment 4 we also observed that birds used the foot opposite to the direction of approach (all birds 85 - 100%; all Sign test p < 0.05; see Table S4 in the Material for details), which also happens to be the foot that is facing away from the wall. This means that while the choice of landing site may change depending on the particular configuration of the landing task, there is a clear relationship between the direction of approach and the foot that is used to make first contact. Landing on this foot would presumably be safer as it would make the bird tip toward the wall, rather than away from it. However, this tendency may also be a consequence of the preference of the birds to approach the perch from an oblique direction, which minimizes the likelihood of the wing making contact with the wall.

One possible interpretation of the results of Experiments 1 and 4 might therefore be that foot preference drives the observed side bias. However, for any given individual, the choice of the direction of approach (and therefore the choice of foot for landing) seems to differ between the two tasks, and also differs from the foot preference determined in Experiment 5. This makes it unlikely that foot preference is the sole determinant of the biases that are exhibited by individual birds.

### Correlation between Experimental Results

In order to further investigate the relationship between the outcomes of Experiments 1,2, 4 and 5 we performed a correlational analysis of the results. The biases displayed by the birds across Experiments 1, 2, 4 and 5 are summarised in Table 2 (for details see Table S5 in the Material). These biases were compared statistically using the Spearman Rank correlation test, the results of which are shown in Table 3 in the form of a correlation matrix. The correlation matrix data indicates no parallels between the individual experiments, suggesting that foot preference alone cannot explain the side biases observed in the individual tasks.

**Table 2:** Summary of biases.

	**Experiment**			
**Bird**	**1**	**2**	**4**	**5**
**Black Hole**	**L**	R	**L**	**R**
**Drongo**	**L**	L	**L**	**R**
**Four**	**R**	**R**	**R**	L
**Milkyway**	**R**	R	**R**	**R**
**Nemo**	**L**	**L**	**R**	**L**
**One**	**L**	**R**	**L**	R
**Rama**	**L**	L	L	R
**Stardust**	**L**	**R**	**R**	R
**Supernova**	**L**	**R**	R/L	**R**
**Three**	**L**	L	**L**	L
**Titan**	**R**	**L**	R	L
**Two**	**L**	L	**R**	**R**

L: Left bias; R: Right Bias; Significant Biases are indicated in bold characters. More detailed results in the form of laterality indices can be found in the Material (Table S5)

**Table 3:** Correlation matrix.

	**Exp. 2**	**Exp. 4**	**Exp. 5**
Exp. 1	0.301	0.121	-0.103
Exp. 2		-0.206	0.276
Exp. 4			-0.393

Correlations between the individual experiments as determined by the Spearman Rank Correlation test.

Another possibility would be that biases are determined by a combination of foot preference and other factors. In order to investigate this we additionally performed a canonical correlation analysis (Table 4), investigating the relationship across multiple tasks, i.e. looking at the outcome of two or more experiments and testing whether they can be used to predict the results of any of the experiments. Again, as shown in Table 4, none of the canonical correlates reaches significance, indicating that the tasks are independent of each other, and therefore suggesting that the biases observed in each of these tasks are not related to each other.

**Table 4:** Canonical correlations between multiple sets of tasks.

**Predictor Sets**			**Lambda**	**Chi**	**DF**	**Sign?**
Exp. 1	Exp. 5		0.608	4.23	4	n.s.
Exp. 2	Exp. 5		0.717	2.83	4	n.s.
Exp. 4	Exp. 5		0.616	4.11	4	n.s.
Exp. 1	Exp. 2	Exp. 5	0.738	2.58	3	n.s.
Exp. 1	Exp. 4	Exp. 5	0.760	2.33	3	n.s.
Exp. 1	Exp. 2	Exp. 4	0.686	3.20	3	n.s.
Exp. 2	Exp. 4	Exp. 5	0.706	2.96	3	n.s.

Predictor sets: sets of tasks used to form the canonical correlate. Lambda: deviation of the canonical correlate from the observed values in the other sets. Chi: test statistic. Sign: indicates whether or not the canonical correlate proved to be a significantly good predictor for the observation sets.

## Discussion

Recent studies have shown that population biases for specific tasks, when they exist, are not consistent across all bird species. For example, even within the class of Australian parrots, there are large species-dependent variations with respect to the eye that is used to view food, as well as the foot that is used to pick it up [7-9]. 

Our study takes this one step further to reveal that even within a given species, birds can display lateralization that varies from task to task, as well as across individuals with respect to a given task. We find that Budgerigars display strong, individually varying lateralization with respect to their choice of perch, landing location, or direction of approach whilst landing, even in very similar tasks. 

As mentioned in the Introduction, most previous studies report the existence of lateralization at the population level. However, there are a few documented instances of lateralization at the individual level. For example, Pigeons display individually varying, and time-varying preferences for the foot that makes first contact when landing on a platform [22]; great tits exhibit individually varying foot preferences while holding or manipulating food [23]; and Japanese jungle crows show individually varying foot preferences while scratching their beak or handling food [24]. Our findings add to this compendium of knowledge by documenting that Budgerigars show individually varying side biases for the choice of landing sites. In particular, Experiment 2, which investigated landings of Budgerigars on a long perch, has interesting parallels with studies of line bisection tasks with humans, which also show variations of bias polarity across individuals e.g. [25].

### Population Bias or Individual Bias?

The results of Experiment 1 (choice between two perches) reveal a strong and statistically significant left-bias in all except three birds, which show a right-bias. This particular experiment may be suggestive of a bias at the population level. However, the results of Experiments 2, 4 and 5 indicate biases that vary with the individual, as well as with the task at hand. This wide variation suggests that, at least with respect to the tasks we have studied, the biases occur at the level of the individual, and not at the population level. Our findings are partly in accordance with a previous study of tree swallows, where no bias was found at the population level in a task in which the birds had to choose to fly through one of two apertures of different sizes [26]. It is noteworthy, however, that their study reported an absence of lateralization at the individual level, although data for individuals was not provided. Brown and Magat [8] compared, across individuals, biases in the eye that is used to view food and the foot that is used to pick it up, in a number of different species. They found striking correlations on an individual basis: an individual that viewed food predominantly with its right eye was more likely to pick it up with its right foot, and vice versa. While our investigation explored a rather different set of tasks, we do not find such correlations in the behaviours that we have studied.

### Task-dependent Changes of Bias Polarity in Individual Birds

Our study also reveals that slight alterations in the task can cause the bias of a particular individual to reverse, i.e. change its polarity. Other studies on birds, so far, indicate that laterality at the individual level, especially in footedness, remains consistent over different tasks. In a study on the great tit, *Parus major*, Vince [23] reports consistent foot preference in two tasks, food holding and string pulling, and in a study on the Japanese jungle crow, *Corvus macrorhynchos*, by Izawa and colleagues [24] similar results were obtained when comparing two tasks, namely food-holding and beak scratching. Previous studies in Budgerigars have so far focused solely on food detection, food handling or manipulation [For example see 7-9,27] - a rather arbitrary choice considering that Budgerigars rarely use their feet for feeding or manipulating objects. All of these studies indicate that Budgerigars display strong lateralization on the individual level, and are suggestive of the absence of population biases.

To our knowledge, there are only two other studies so far that have reported direct evidence for a task-dependent reversal of bias. Waters and Denenberg [28] found that mice exhibited a change in bias at the population level, when a simple reaching task was modified slightly. Hook and Rogers [29] investigated stability of biases in marmosets over a variety of reaching tasks. They found that the biases displayed by each individual remained the same for all but one task. 

Even though there is no direct correlation between the individual biases displayed in Experiment 1 and Experiment 4, it is intriguing that the birds, regardless of which side they choose, consistently use the outermost foot to make first contact with the perch. Doing so may allow the birds to achieve greater stability – in each case allowing the bird to tip toward the wall, rather than away from it in case of an emergency.

### Are Individual Biases Preserved when Animals Interact in Larger Groups?

Studies of biases in larger groups, so far, are limited to the reactions of chicks [5] and quails [6] towards mates and strangers, indicating, at least indirectly, that animals do not display individually differing biases when their behaviour is observed in large groups. While only indirectly related to our observations, this is in accordance with our finding that the preference that is displayed by a Budgerigar when it is tested on its own is no longer observed when the bird is paired up with another individual. This is intriguing, as it is in stark contrast with the central hypothesis explaining the evolutionary origins of population biases in social animals, i.e. that individual biases could direct a group of animals to move in specific directions [13]. While our study may not be sufficient to reject this hypothesis, it underscores the need to further explore the relationship between the behaviour of individuals and that of a population.

Our study suggests that Budgerigars display indications of a population bias with respect to one particular task, namely choosing between a left-hand perch and a right-hand perch. However, in other tasks, some of which are variants or more complex versions of the same task, the birds display biases that are strongly dependent upon the individual, and upon the task at hand. Furthermore, the individual biases vanish completely when one bird is paired up with another. Thus, lateralization of behaviour in Budgerigars (and perhaps other animals) is likely to be more complex and subtle than hitherto supposed.

## Supporting Information

Figure S1 **Number of landings in each of the segments for Experiment 2.**
(DOCX)

Table S1 **Average landing position and standard deviation for Experiment 2.**
(DOCX)

Table S2 **Average landing position of individual birds in Experiment 3.**
(DOCX)

Table S3 **Foot used when landing on the perches in Experiment 1.**
(DOCX)

Table S4 **Foot used when landing on the perch in Experiment 4.**
(DOCX)

Table S5 **Laterality indices.**
(DOCX)
