# Supplementary material for: Behavioural Lateralization in Budgerigars Varies with the Task and the Individual
Source: PLoS One. 2013 Dec 6;8(12):e82670. doi: 10.1371/journal.pone.0082670 (PMC3855779; doi:10.1371/journal.pone.0082670)
Supplement: Figure S1 — Number of landings in each of the segments for Experiment 2. (DOCX) [file pone.0082670.s001.docx]

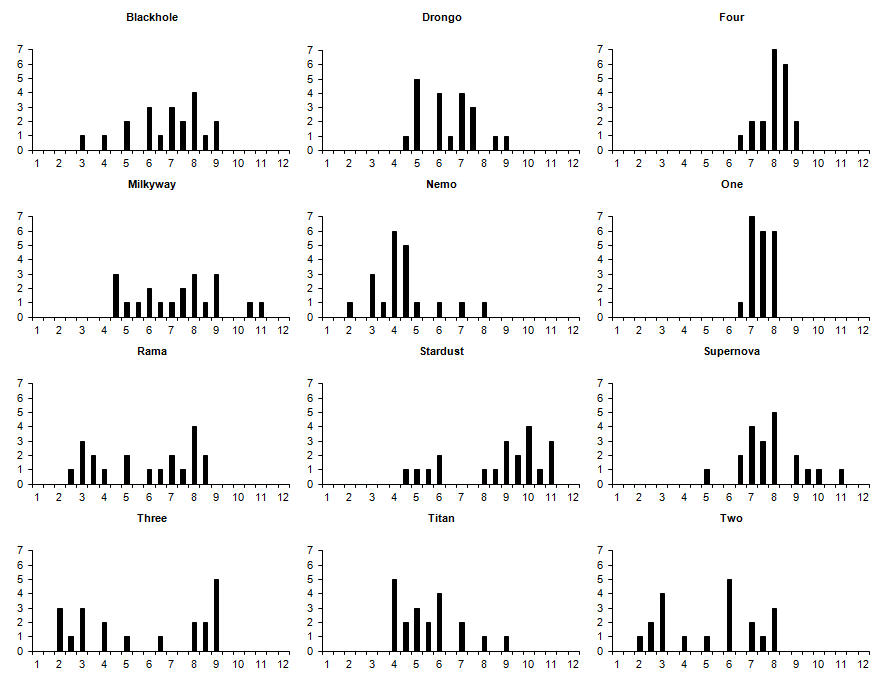


Figure S1. Number of landings in each of the twelve segments for Experiment 2. Segment 1 is the leftmost segment and 12 the segment furthest to the right.
