# Supplementary material for: Behavioural Lateralization in Budgerigars Varies with the Task and the Individual
Source: PLoS One. 2013 Dec 6;8(12):e82670. doi: 10.1371/journal.pone.0082670 (PMC3855779; doi:10.1371/journal.pone.0082670)
Supplement: Table S1 — Average landing position and standard deviation for Experiment 2. (DOCX) [file pone.0082670.s006.docx]

**Table S1. Average landing position and standard deviation for Experiment 2.**

| **Bird** | **Average** | **St. Dev.** |
| --- | --- | --- |
| **Black Hole** | **6.8** | **1.6** |
| **Drongo** | **6.4** | **1.3** |
| **Four** | **8.0** | **0.7** |
| **Milkyway** | **7.3** | **1.9** |
| **Nemo** | **4.4** | **1.4** |
| **One** | **7.4** | **0.5** |
| **Rama** | **5.8** | **2.2** |
| **Stardust** | **8.7** | **2.1** |
| **Supernova** | **7.9** | **1.4** |
| **Three** | **5.8** | **2.9** |
| **Titan** | **5.5** | **1.4** |
| **Two** | **5.2** | **2.1** |

The midpoint of the perch corresponds to a landing position of 6.5.
