# Supplementary material for: Behavioural Lateralization in Budgerigars Varies with the Task and the Individual
Source: PLoS One. 2013 Dec 6;8(12):e82670. doi: 10.1371/journal.pone.0082670 (PMC3855779; doi:10.1371/journal.pone.0082670)
Supplement: Table S2 — Average landing position of individual birds in Experiment 3. (DOCX) [file pone.0082670.s002.docx]

**Table S2. Average landing position of individual birds in Experiment 3.**

|  | **Release Position** | |
| --- | --- | --- |
| **Bird** | **Left** | **Right** |
| **Black Hole** | 1.9 | 5.6 |
| **Drongo** | 5.3 | 8.6 |
| **Four** | 6.0 | 10.6 |
| **Milkyway** | 9.2 | 9.9 |
| **Nemo** | 5.0 | 8.1 |
| **One** | 4.7 | 6.9 |
| **Rama** | 2.3 | 8.0 |
| **Stardust** | 3.2 | 7.6 |
| **Supernova** | 4.8 | 7.4 |
| **Three** | 7.9 | 4.8 |
| **Titan** | 7.3 | 9.2 |
| **Two** | 8.1 | 5.4 |

12 birds showed clear tendencies to fly towards the left when released from the left and towards the right when released from the right. Except for 3 birds which preferred one side of the perch, all birds chose the left half of the perch when released from the left and the right half of the perch when released from the right.
