# Supplementary material for: Behavioural Lateralization in Budgerigars Varies with the Task and the Individual
Source: PLoS One. 2013 Dec 6;8(12):e82670. doi: 10.1371/journal.pone.0082670 (PMC3855779; doi:10.1371/journal.pone.0082670)
Supplement: Table S4 — Foot used when landing on the perch in Experiment 4. (DOCX) [file pone.0082670.s004.docx]

**Table S4.** **Foot use when landing on the perch in Experiment 4.**

| **Bird** | **N** | **Same** | **Opposite** | **%** | **Sign** |
| --- | --- | --- | --- | --- | --- |
| **Black Hole** | 17 | 2 | 15 | 88% | ** |
| **Drongo** | 20 | 1 | 19 | 95% | ** |
| **Four** | 20 | 0 | 20 | 100% | ** |
| **Milkyway** | 18 | 0 | 18 | 100% | ** |
| **Nemo** | 20 | 0 | 20 | 100% | ** |
| **One** | 18 | 0 | 18 | 100% | ** |
| **Rama** | 19 | 0 | 19 | 100% | ** |
| **Stardust** | 15 | 0 | 15 | 100% | ** |
| **Supernova** | 19 | 0 | 19 | 100% | ** |
| **Three** | 20 | 0 | 20 | 100% | ** |
| **Titan** | 13 | 2 | 11 | 85% | * |
| **Two** | 20 | 0 | 20 | 100% | ** |
| **Overall** | 12 | 0 | 12 | 100% | ** |

N: number of landings analysed (excluding those in which the landing foot could not be clearly discerned from the video images); Same/Opposite: number of times the foot was on the same side on which the bird landed, or on the opposite side; % frequency of the observed preference; Sign: significance level, as determined by the Sign Test (with **: p<0.01 and *:p<0.05).
