# Supplementary material for: Behavioural Lateralization in Budgerigars Varies with the Task and the Individual
Source: PLoS One. 2013 Dec 6;8(12):e82670. doi: 10.1371/journal.pone.0082670 (PMC3855779; doi:10.1371/journal.pone.0082670)
Supplement: Table S5 — Laterality indices. (DOCX) [file pone.0082670.s005.docx]

**Table S5.** **Laterality indices.**

|  | **Experiment** | | | |
| --- | --- | --- | --- | --- |
| **Bird** | **1** | **2** | **4** | **5** |
| **Blackhole** | -60 | 26 | -90 | 90 |
| **Drongo** | -100 | -5 | -90 | 70 |
| **Four** | 90 | 100 | 90 | -40 |
| **Milkyway** | 80 | 26 | 100 | 50 |
| **Nemo** | -100 | -90 | 100 | -60 |
| **One** | -80 | 100 | -100 | 40 |
| **Rama** | -80 | -5 | -30 | 30 |
| **Stardust** | -100 | 50 | 60 | 25 |
| **Supernova** | -70 | 89 | 0 | 100 |
| **Three** | -60 | -5 | -90 | -20 |
| **Titan** | 80 | -60 | 30 | -30 |
| **Two** | -90 | -40 | 80 | 60 |

The laterality index is defined as: [(R-L)/(R+L)] * 100
